# Dependable modulation classifier explainer with measurable explainability

**DOI:** 10.3389/fdata.2022.1081872

**Published:** 2023-01-09

**Authors:** Gaurav Duggal, Tejas Gaikwad, Bhupendra Sinha

**Affiliations:** Reliance Industries, Mumbai, India

**Keywords:** visualization, constellation diagram, modulation classification, explainability, fair AI

## Abstract

The Internet of Things (IoT) plays a significant role in building smart cities worldwide. Smart cities use IoT devices to collect and analyze data to provide better services and solutions. These IoT devices are heavily dependent on the network for communication. These new-age networks use artificial intelligence (AI) that plays a crucial role in reducing network roll-out and operation costs, improving entire system performance, enhancing customer services, and generating possibilities to embed a wide range of telecom services and applications. For IoT devices, it is essential to have a robust and trustable network for reliable communication among devices and service points. The signals sent between the devices or service points use modulation to send a password over a bandpass frequency range. Our study focuses on modulation classification performed using deep learning method(s), adaptive modulation classification (AMC), which has now become an integral part of a communication system. We propose a dependable modulation classifier explainer (DMCE) that focuses on the explainability of modulation classification. Our study demonstrates how we can visualize and understand a particular prediction made by seeing highlighted data points crucial for modulation class prediction. We also demonstrate a numeric explainability measurable metric (EMM) to interpret the prediction. In the end, we present a comparative analysis with existing state-of-the-art methods.

## 1. Introduction

**Figure 1:**
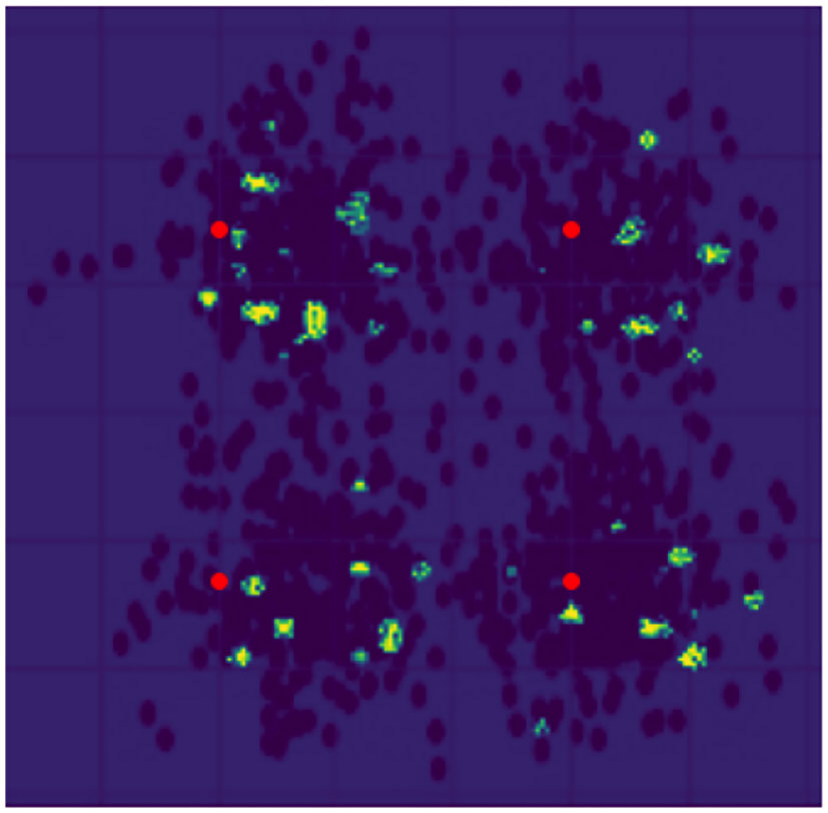
Result: 4PSK. Red dots are ideal points. Yellow and Green dots represent an explanation of the prediction made by the modulation classifier.

Industries today are influenced by AI capabilities, and IoT industries are no exception. May it be data transmission capabilities, cost-effective network roll-out or operation, system performance, customer service, or any other telecom application (Balmer et al., 2020), AI has marked its presence. A study (AI in 5G) suggests that Telecom, IoT, and AI will play a significant role in market disruptions in the coming decades (Balmer et al., 2020). IoT devices in the smart city setup depend on the network underneath to work flawlessly. The smart city network uses a telecom setup to communicate and send the data between the nodes and service points. For every signal getting transmitted to different locations, a lot of computation, processing, encoding, and transformation happens in the back end. The transmission uses modulation schemes to modulate the signal to send data to longer distances, making it prone to noise and reducing power requirements at the transmitter end. AI-based modulation classifiers are used to correctly and automatically identify the signal's modulation scheme. The involvement of AI in this area has massive utilization for high-dimensional data, which is also highly critical. If not handled appropriately, it may cause harm to the system and its users and cause a financial impact. Rana et al. (2022) shed light on the dark side of AI. The authors discuss how insufficient efforts, recognized risks, and unclear basis for decisions can lead to a firm's operational inefficiency and bring a competitive disadvantage. They proposed a research model that captures such components that can cause fallback to a firm and analyses risk factors and negative performance. Thus, it becomes equally important to keep track of what AI is trying to provide. Is it the same thing we as a developer or a user want, or is it merely a combination of correlations that luckily gives good accuracy and can predict a significant section of test data? Thus, the models integrated into systems responsible for making critical business decisions must be explainable and interpretable. It would help make a smart city network fair and trustable. In this study, we demonstrate explanations that can further be used to measure the model's effectiveness, i.e., having an interpretable and explainable AI. Explainability term refers to the extent to which the output of the model can be explained, and interpretability refers to the extent to which the model's output can be interpreted by its user. When it comes to having a fair model, interpretability and explainability hold important roles (Murdoch et al., 2019). Our article presents a machine-learning application in the field of communication. It describes the use of convolutional neural network (CNN), a part of deep learning (DL) in modulation classification using constellation points (Kojima et al., 2019), a crucial stage in all communications systems. It relies on a large volume amount of data to have a good-performing model, and this data availability may be limited in communications systems. We have generated our data using Labview software which gives out constellation points with a wide range of SNR levels. Notably, we have used the gradient-based methodology GradCAM++ (Chattopadhyay et al., 2017) to visualize the predictions made by the model in the form of a heat map. Brighter the pixel, the more critical information it holds. Figure 1 shows the explainability of the 4PSK modulated signal. The red points are the ideal points of the signal, and the highlighted points are identified as crucial points causing correct prediction of the modulation class.

### 1.1. Motivation

The prime motivation for our study is to have a fair and trustable AI system when we discuss a smart network system, especially for applications where AI is making critical business decisions. It can be achieved by having explainable and interpretable AI systems (models). The explainable term refers to a model whose output can be explained by some means. The interpretable discusses more about the scale to which humans understand explanations for critical areas like medicine, law, defense, finance, and some telecommunications areas. AI should be accountable for the decisions/ predictions made.

### 1.2. Contribution

We proposed a dependable modulation classifier explainer (DMCE) with explainability measurable metric (EMM). In this study, dependable refers to a fair and bias-free model that can be achieved by having the model explainable and interpretable. In our study, we proposed explanations for correctly and incorrectly classified classes and the explanation scores for each class. The explanations are presented in the form of heat maps that highlight data points crucial for that specific class. Our explainability score measures the relevancy of identified critical data points. The explainability score ranges from 0 to 1, where 1 is the maximum and ideal case.

## 2. Previous work

Automatic modulation classification, constellation diagrams, and gradient-based methods for visualization of the model predictions are core components of our study. In the following section, we discussed details of the works that we have inherited.

### 2.1. Classification using a standard machine learning approach

A constellation diagram is a 2-D representation of a modulated signal by mapping signal samples into scattering points on a complex plane (Zhendong et al., 2013; Kumar et al., 2020). This study discusses modulation recognition algorithms for M-QAM signals used in IoT devices and digital equipment. In this study, the authors used k-means clustering to perform modulation classification.

### 2.2. Previous DL methods for modulation classification

Automatic Modulation Classification (AMC) is used to identify modulation types effectively and automatically. Wang et al. (2018) demonstrated that DL-based AMC works effectively in single-input-single-output systems but is scarcely explored for multiple-input-multiple-output (MIMO) systems. This study demonstrates that CNN-based AMC has the highest correct classification probability. Kojima et al. (2019) presented methods representing modulated signals in various data formats with grid-like topology for the CNN model. The authors demonstrated a considerable performance advantage of DL based approach for modulation classification. Wang et al. (2018) presented a constructed multi-layer hybrid machine learning network for classifying seven types of signals in different modulation by extracting modulated signal features using support vector machines and naive Bayes to accomplish automatic modulation classification. The simulated results demonstrated the accomplishments of their approach with significant improvement in classification accuracy. Huang et al. (2020) present a method to visualize different LSTM and CNN models for radio modulation classifiers. The authors also demonstrated different hyper-parameter settings for extracting radio features relating to modulation reference points.

### 2.3. Visualization using gradient-based methods

GradCAM (Selvaraju et al., 2017) and GradCAM++ (Chattopadhyay et al., 2017) are explanation techniques for CNN-based implementations. It gives a heatmap of predictions made by the model and highlights the pixel critical for a particular class prediction. It uses the target class' gradients and the convolution network's final layer to produce the desired output.

## 3. Methodology

**Figure 2:**
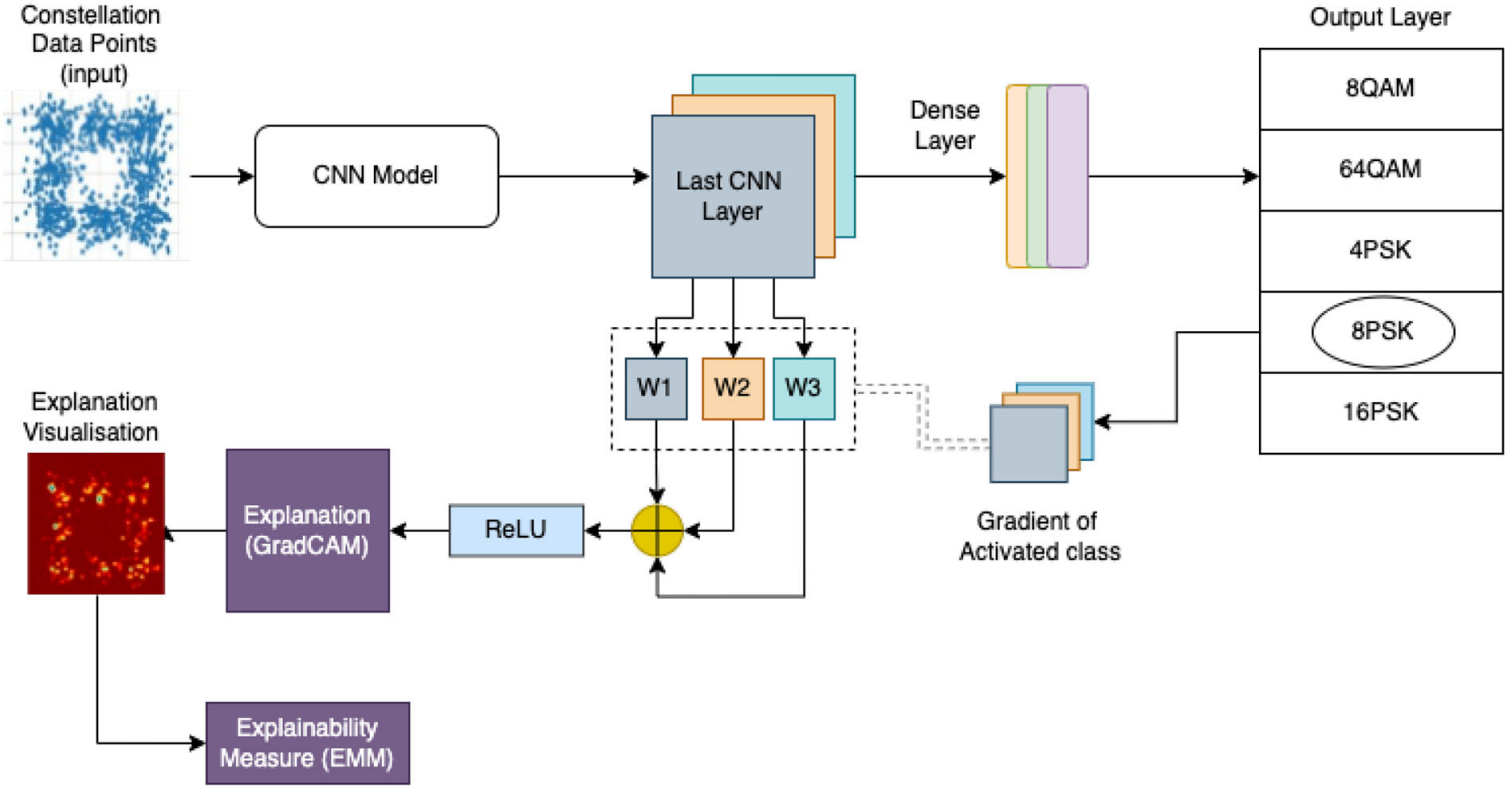
Our proposed system architecture. It takes constellation data points as image input. This image is fed to a trained CNN whose last convolutional neural network (CNN) layer is used to obtain an explanation shown as explanation visualization.

**Figure 3:**
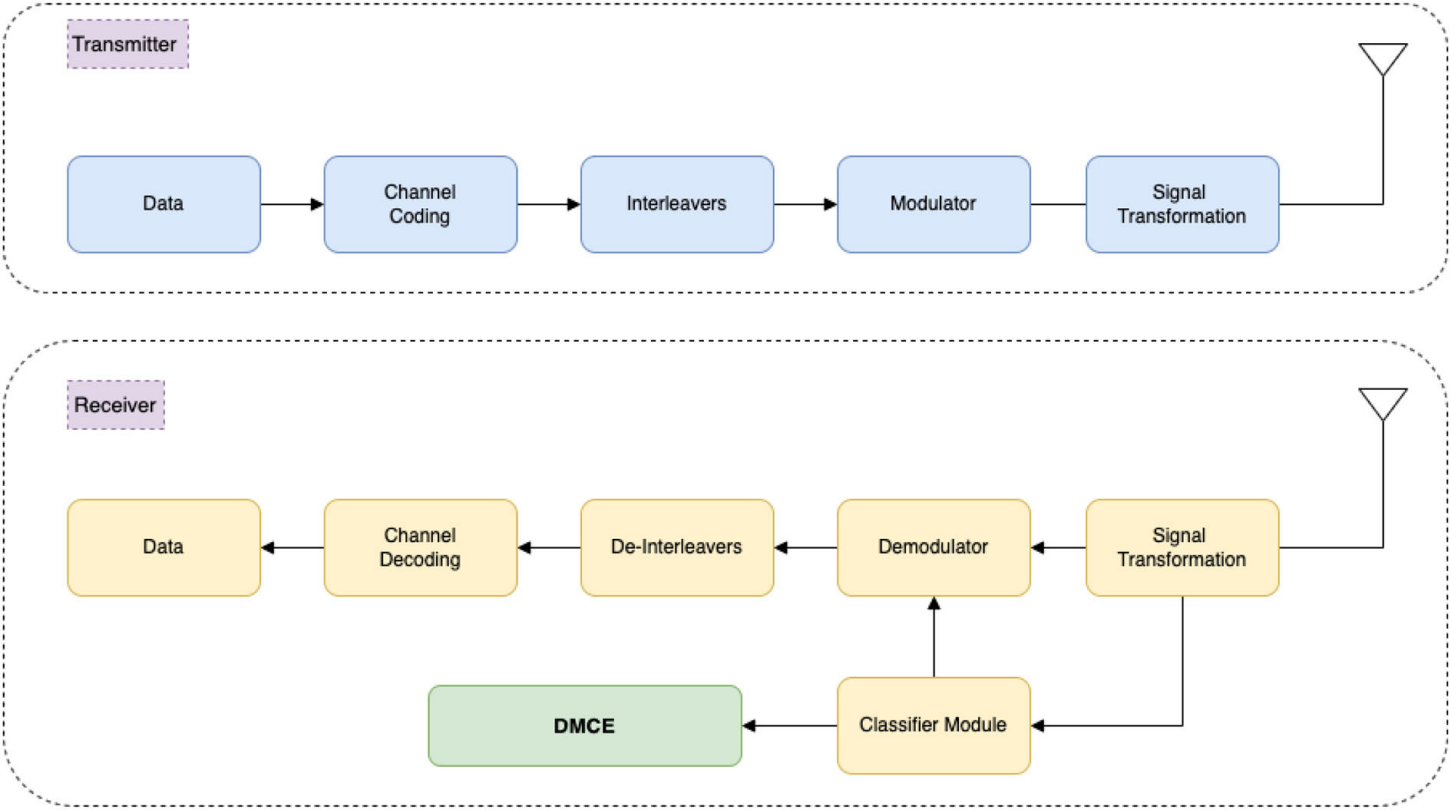
A complete overview of end-to-end pipeline of a standard communication system and understanding of locating our DMCE block in the communication pipeline.

As shown in Figure 2, the system has three main categories: transmission of the signal, reception of the signal, and the DMCE block. The first category is the transmission of an input signal with additive white Gaussian noise (AWGN). This stage is responsible for the modulation and transmission of the signal. The second category is the modulation signal's reception and identifying modulation class using our AMC. This stage is responsible for receiving, denoising, identifying the modulation scheme, and demodulating the signal. The third and the last category is our proposed DMCE. Figure 3 shows the DMCE system diagram demonstrating the steps followed for classification and explanation.

Our work is divided into four stages: conversion of signal into constellation points, predicting modulation scheme class, explaining the modulation output, and generating the relevancy measure score (EMM). The signal received is converted into a set of constellation points and then into an image. This image is fed to the classifier module AMC to identify the modulation class. After identifying the modulation class, the gradient of the final CNN layer for the activated class is obtained in the backpropagation process. This gradient of the activated class, i.e., the gradient of the last CNN layers of the activated class, is further operated using global average pooling. After the pooling operation, summation for R, G, and B matrices is done, post which the Re-LU activation function is applied. This operation will give a heat map of the activated class. The heat map is the explanation visualization for the input image, and this image is further given to the explainability measure block to obtain the EMM score and check the relevancy measure. Algorithm 1 gives a detailed approach of entire process.

**Algorithm 1:**
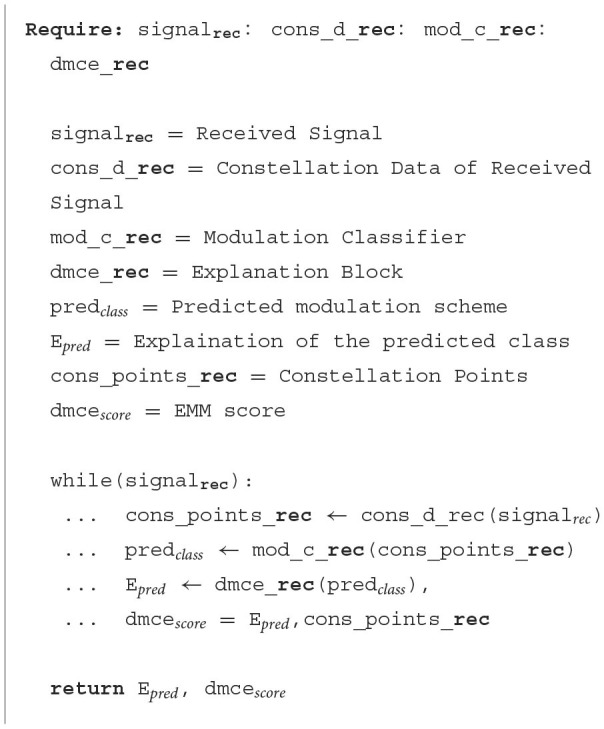
Policy for getting explanations for the receiver's input.

## 4. Implementation

### 4.1. Signal conversion

The signal is in the form of an electromagnetic wave. This electromagnetic signal is received *via* antennas and converted into an electronic signal using a transducer. This electronic signal is then used to generate constellation data points. The constellation data points are converted into an image which is then given to our CNN model to identify the modulation scheme used during transmission.

### 4.2. Predicting modulation scheme

The constellation points image of the input signal obtained from the previous stage are fed to our CNN-based AMC to identify the modulation scheme. The AMC will give the modulation scheme and its confidence score for the predicted class.

### 4.3. Explaining the results

**Figure 4:**
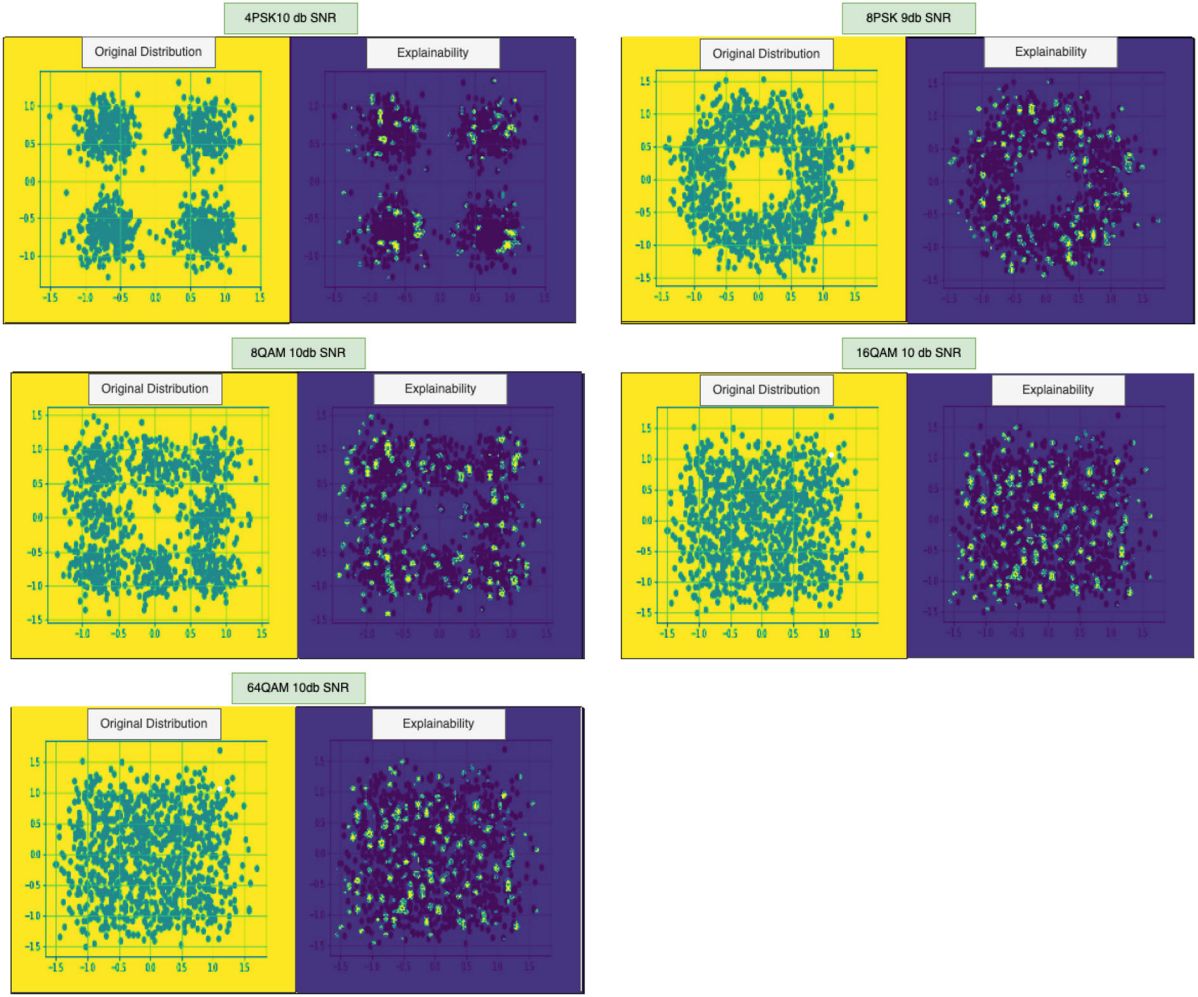
Explainability Results: (In order left to right) 4PSK, 8PSK, 8QAM, 10QAM, and 64QAM. The yellow colored box represents the actual constellation points of a given class, and the blue box represents a visual explanation of predictions for the respective sample.

In this stage, we obtain an explanation of the modulation class prediction done at the previous stage, i.e., AMC. Here, we extract the weights of the final convolutional layer of the AMC and obtain gradients of those layers followed by global average pooling. These layers are then given to Rectified Linear Unit (ReLU) activation function to generate heatmaps (Figure 4). These heatmaps are then combined with the original data image to identify actual data points responsible for predicting a particular class.

### 4.4. Explainability measurable metric

Explanation Measurable Metric is a crucial metric to identify the correctness of explainability. The correctness of EMM scores is obtained by measuring an aggregate distance between the data points' heat map and the ideal data points (shown as a red dot in Figure 1 of the modulation scheme). This score is between 0 and 1, and a score above 0.5 gives a good prediction model. Analysis of this is required to justify the explanations so that a user can trust the explanations given by our AMC.

### 4.5. Dataset

We have created this dataset using LabView software. The constellation data is obtained for 4PSK, 8PSK, 8QAM, 16QAM, and 64QAM with SNR ranging from 0 to 10db.

### 4.6. Training and hyper-parameters

A 9-level 2D-CNN-based architecture is used for building our automatic modulation classification (Kumar et al., 2020). 2D CNN was used to reduce the computation as we are concerned only with the position of the data points. A 9-level CNN network was a result of experimentation and hyper-parameter tuning. We have generated constellation points for the following five classes for our implementation: 64 QAM, 4PSK, 16QAM, 8 PSK, and 8 QAM with a range of SNR values from 0 to 10 db. A standard communication network with a signal SNR above 20 db is considered good quality. These classes are considered to cover phase shift keying and amplitude modulation schemes and examine more straightforward and complex modulation schemes together. The CNN model is trained on around 2–2.5 k samples in each class with an SNR range from 0 to 10 db, where 0db SNR is the worst. We have obtained 74.6% validation accuracy for classification. We have used the GradCAM (Chattopadhyay et al., 2017) method to visualize the critical data points for the activated class. As shown in Figure 3, we have taken the convolutional network's last layer of the predicted class and used the last CNN layer weights to obtain the explainability. Details are discussed in Sections 3, 4.

### 4.7. Error-analysis

Error-Analysis for the modulation classifier is given below. The first column, “Possible Hypothesis for Error,” represents the cases where the miss-classification occurred at most. These are categorized into 4 sections, SNR > 8 db, SNR > 5 db, SNR > 3 db, and SNR > 0 db. With the percentage of total errors, we can see that the errors or miss-classification happen the most when SNR drops below 5 db. The permissible value of SNR in the real-time scenario is 20–30 db.

### 4.8. Comparative analysis

The existing methods focus on identifying the best-performing approaches that can be used for automatic modulation classification. On top of it, existing methods (Peng et al., 2019; Huang et al., 2020) have performed an analytical operation to visualize the data points and predictions of the AMCs. These methods do not demonstrate any approach that explains the reason for predicting the activated class. They neither have any metric to justify the correctness of their respective approaches. Moreover, the interpretability of the proposed visualization in these works(s) is one of the demerits.

In our work, we have overcome these demerits. We have proposed and demonstrated explanations for predictions by our AMC. The highlighted data points can be compared to the positions of the ideal constellation points, and an EMM score also gives insights into the explanation's correctness.

## 5. Experiments and results

Our error analysis, as shown in Table 1, and explainability measurable metric, as shown in Table 2, shows that the explainability helps us understand the weak areas where the model is not performing well and also gives a numerical analysis of model performance for different classes. Furthermore, our results are easy to interpret thus, helps to provide transparency for the AI models (Figure 4). We found minor implementations discussing interpretability and explainability modulation classification, but in comparison to these methods, our method demonstrates the credibility of explained critical data points with the help of EMM.

**Table 1:** Error-Analysis for our CNN-based Automatic Modulation Classifier (AMC).

**Possible hypothesis for error (Out of 100)**						**Percentage of total errors (%)**				
**Modulation class count ->**	**4 PSK**	**8 PSK**	**8 QAM**	**16 QAM**	**64 QAM**	**4 PSK**	**8 PSK**	**8 QAM**	**16 QAM**	**64 QAM**
With SNR >8 db	12	16	20	17	23	10.6	14.8	18.5	15.80	20.53
With SNR >5 db	25	34	32	39	42	22.12	31.48	29.62	36.44	37.5
With SNR >3 db	38	44	51	63	63	33.62	40.7	47.22	58.87	56.25
With SNR >0 db	44	52	47	66	73	38.90	48.14	43.51	61.60	65.17
Total samples	113	108	108	107	112					

The performance is tested for different SNR values ranging from 0 to 10 db. It is observed that the AMC is performing well for high SNR values and less complex modulation classes. The comparison is done for randomly selected 100 samples.

**Table 2:** The error analysis and reasoning *via* explainability measurable metric (EMM).

	**Samples correctly classified**		**Samples incorrectly classified**	
**Modualation** ** Class**	**SNR Ratio** ** (Top 10)**	**Explanation Score** ** (Avg. Top 10)**	**SNR Ratio** ** (Top 10)**	**Explanation Score** ** (Avg. Top 10)**
4 PSK	10 db	0.74	1 db	0.34
8 PSK	10 db	0.61	3 db	0.26
8 QAM	10 db	0.68	3 db	0.19
16 QAM	10 db	0.54	3 db	0.17
64 QAM	10 db	0.52	5 db	0.11

The evaluation is done on five classes, namely 4 PSK, 8 PSK, 8 QAM, 16 QAM, and 64 QAM. For a good SNR ratio, the classification is successful, and it is fairly supported by the explanation score (EMM). Similarly, the explanation score is comparatively a smaller value for the mis-classification scenario.

## 6. Future work

Our work considers demodulation block, which uses deep learning techniques for AMC. The explainability and interpretability components are much-needed components of any system where AI plays a crucial role in making decisions. The extension of this work will be a continuous learning and improvement system for these models by taking feedback from the explanations and EMM to further optimize the models by performing hyper-parameter selection and tuning.

## 7. Conclusion

We propose a DMCE that helps to understand predictions by modulation classifier in a smart city network of connected devices. DMCE provides a visualization-based explanation by highlighting data points crucial for a modulation class prediction. We have added an EMM that gives insights about working/ failing cases for the model by providing numerical analysis, thus enhancing the explainability. The system's error analysis helps us to get better insights into areas where the modulation classifier needs to be fixed.

## Data availability statement

The original contributions presented in the study are included in the article/supplementary material, further inquiries can be directed to the corresponding authors.

## Author contributions

All authors listed have made a substantial, direct, and intellectual contribution to the work and approved it for publication.

## References

[B1] Balmer R. E. Levin S. L. Schmidt S. (2020). Artificial intelligence applications in telecommunications and other network industries. Telecommun. Policy 44, 101977. 10.1016/j.telpol.2020.101977

[B2] Chattopadhyay A. Sarkar A. Howlader P. Balasubramanian V. N. (2017). Grad-cam++: generalized gradient-based visual explanations for deep convolutional networks. CoRR, abs/1710.11063. 10.1109/WACV.2018.00097

[B3] Huang L. Zhang Y. Pan W. Jinyin C. Qian L. Wu Y. (2020). Visualizing deep learning-based radio modulation classifier. IEEE Trans. Cogn. Commun. Network. 7, 47–58. 10.1109/TCCN.2020.3048113

[B4] Kojima S. Maruta K. Ahn C.-J. (2019). Adaptive modulation and coding using neural network based snr estimation. IEEE Access 7, 183545–183553. 10.1109/ACCESS.2019.2946973

[B5] Kumar Y. Sheoran M. Jajoo G. Yadav S. K. (2020). Automatic modulation classification based on constellation density using deep learning. IEEE Commun. Lett. 24, 1275–1278. 10.1109/LCOMM.2020.2980840

[B6] Murdoch W. J. Singh C. Kumbier K. Abbasi-Asl R. Yu B. (2019). Definitions, methods, and applications in interpretable machine learning. Proc. Natl. Acad. Sci. U.S.A. 116, 22071–22080. 10.1073/pnas.1900654116 31619572 PMC6825274

[B7] Peng S. Jiang H. Wang H. Alwageed H. Zhou Y. Sebdani M. M. . (2019). Modulation classification based on signal constellation diagrams and deep learning. IEEE Trans. Neural Netw. Learn. Syst. 30, 718–727. 10.1109/TNNLS.2018.2850703 30047904

[B8] Rana N. P. Chatterjee S. Dwivedi Y. K. Akter S. (2022). Understanding dark side of artificial intelligence (ai) integrated business analytics: assessing firm's operational inefficiency and competitiveness. Eur. J. Inf. Syst. 31, 364–387. 10.1080/0960085X.2021.1955628

[B9] Selvaraju R. R. Cogswell M. Das A. Vedantam R. Parikh D. Batra D. (2017). “Grad-cam: visual explanations from deep networks *via* gradient-based localization,” in 2017 IEEE International Conference on Computer Vision (ICCV) (Venice: IEEE), 618–626.

[B10] Wang F. Huang S. Wang H. Yang C. (2018). Automatic modulation classification exploiting hybrid machine learning network. Math. Prob. Eng. 2018, 1–14. 10.1155/2018/6152010

[B11] Zhendong C. Weining J. Changbo X. Min L. (2013). “Modulation recognition based on constellation diagram for M-QAM signals,” in 2013 IEEE 11th International Conference on Electronic Measurement & Instruments, Vol. 1 (Harbin: IEEE), 70–74.

